# *Mariprofundus ferrooxydans* PV-1 the First Genome of a Marine Fe(II) Oxidizing *Zetaproteobacterium*

**DOI:** 10.1371/journal.pone.0025386

**Published:** 2011-09-23

**Authors:** Esther Singer, David Emerson, Eric A. Webb, Roman A. Barco, J. Gijs Kuenen, William C. Nelson, Clara S. Chan, Luis R. Comolli, Steve Ferriera, Justin Johnson, John F. Heidelberg, Katrina J. Edwards

**Affiliations:** 1 Geomicrobiology Group, Department of Earth Sciences, University of Southern California, Los Angeles, California, United States of America; 2 Bigelow Laboratory for Ocean Sciences, West Boothbay Harbor, Maine, United States of America; 3 Department of Biological Sciences, Marine Environmental Biology Section, University of Southern California, Los Angeles, California, United States of America; 4 Department of Biotechnology, Delft University of Technology, Delft, The Netherlands; 5 Department of Marine Chemistry and Geochemistry, Woods Hole Oceanographic Institution, Woods Hole, Massachusetts, United States of America; 6 Life Sciences Division, Lawrence Berkeley National Laboratory, Berkeley, California, United States of America; 7 J. Craig Venter Institute, San Diego, California, United States of America; University of Minnesota, United States of America

## Abstract

*Mariprofundus ferrooxydans* PV-1 has provided the first genome of the recently discovered *Zetaproteobacteria* subdivision. Genome analysis reveals a complete TCA cycle, the ability to fix CO_2_, carbon-storage proteins and a sugar phosphotransferase system (PTS). The latter could facilitate the transport of carbohydrates across the cell membrane and possibly aid in stalk formation, a matrix composed of exopolymers and/or exopolysaccharides, which is used to store oxidized iron minerals outside the cell. Two-component signal transduction system genes, including histidine kinases, GGDEF domain genes, and response regulators containing CheY-like receivers, are abundant and widely distributed across the genome. Most of these are located in close proximity to genes required for cell division, phosphate uptake and transport, exopolymer and heavy metal secretion, flagellar biosynthesis and pilus assembly suggesting that these functions are highly regulated. Similar to many other motile, microaerophilic bacteria, genes encoding aerotaxis as well as antioxidant functionality (e.g., superoxide dismutases and peroxidases) are predicted to sense and respond to oxygen gradients, as would be required to maintain cellular redox balance in the specialized habitat where *M. ferrooxydans* resides. Comparative genomics with other Fe(II) oxidizing bacteria residing in freshwater and marine environments revealed similar content, synteny, and amino acid similarity of coding sequences potentially involved in Fe(II) oxidation, signal transduction and response regulation, oxygen sensation and detoxification, and heavy metal resistance. This study has provided novel insights into the molecular nature of *Zetaproteobacteria*.

## Introduction

### *Zetaproteobacteria*

*Zetaproteobacteria* are proposed as a novel class of *Proteobacteria* that were first discovered at iron-rich low temperature hydrothermal vents of the Loihi Seamount, Hawaii [1]. Biogenically formed iron oxide mats that cover the seafloor around the seamount are dominated by *Zetaproteobacteria* [2]. *M. ferrooxydans* PV-1 is a representative of a cluster of related isolates that share in common the production of an Fe-oxyhydroxide encrusted helical stalk, and an apparent obligate requirement for ferrous iron (Fe(II)) as an energy source. 16S diversity of the *Zetaproteobacteria* class appears to be high [3], however, all known strains have the ecological and biogeochemically important trait of Fe(II) oxidation, biomineral and iron mat formation in common (*e.g.* [4]). Other related iron-oxidizing *Zetaproteobacteria* have been identified using cultivation-independent techniques at widely distributed sites in deep-sea environments: these include the Red Sea, the Guaymas basin, the Cleft segment hydrothermal system off the coast of Oregon, the Mariana Trench in the Western Pacific, microbial mats from NW Eifuku Volcano along the Marian Island Arc, the South Tonga Arc, and the Cleft Segment of the Juan de Fuca Ridge [1], [5], [6], [7], [8], [9]. Recently, *Zetaproteobacteria* have also been found associated with deep oceanic crustal boreholes in the western Pacific [10] and coastal environments in the eastern United States [11]. Despite the apparent global distribution and biogeochemical importance of the *Zetaproteobacteria*, there has been no genetic or biochemical data on this class; the PV-1 genome thus provides the first molecular insights into potential mechanisms employed by this group to succeed in the deep ocean.

### *Mariprofundus ferrooxydans*

**Figure 1:**
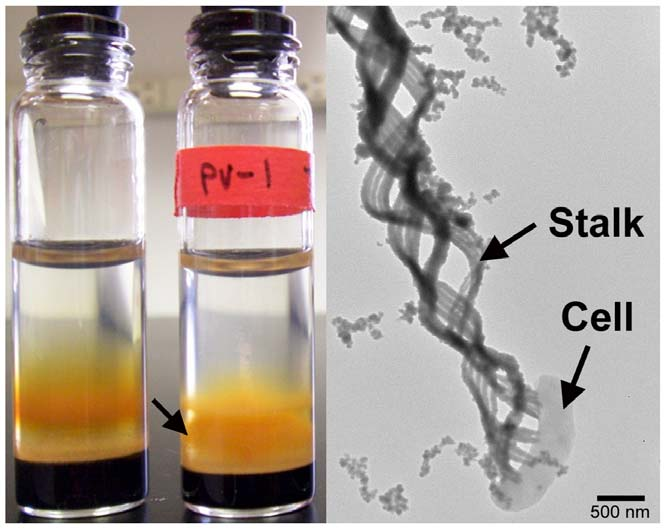
PV-1 cultures. Left: the bottle on the right contains a PV-1 culture in log phase showing orange biological iron oxide precipitates, the left bottle contains the uninoculated control; right: Transmission Electron Microscopy (TEM) picture of a PV-1 cell with twisted stalk made of iron oxides and organic matrix.

Cells of *M. ferrooxydans* are gram-negative, motile curved rods [1]. During its cell cycle, *M. ferrooxydans* alternates between a free-living, often motile stage, and a stage where cells excrete highly structured stalks, primarily composed of iron oxyhydroxides and an organic matrix (Fig. 1) [12]. In the model proposed by [12], stalks direct iron oxide formation, preventing engulfment of the cell by solid phase iron minerals by positioning cells in the dynamic gradients of Fe(II) and O_2_. As noted above, Fe oxide filaments similar to those made by PV-1 have been found broadly in the deep ocean (*e.g.* Axial Volcano, Juan de Fuca Ridge, Vailul'u Volcano, and Loihi) [12], [13], [14].

PV-1 is an obligate chemolithoautotroph that oxidizes reduced Fe from a variety of substrates at pH 5.5–7.2 (*e.g.* FeS, FeCO_3_, FeCl_2_, Fe(NH_4_)_2_(SO_4_)_2_, FeSO_4_, Fe^0^). Oxygen serves as the only electron acceptor and cells are aerotactic [1]. Though Fe(II) oxidizing bacteria (FeOB) have also been isolated from freshwater environments, *e.g.* [2], [15], [16], [17], [18], [19], [20], [21], [22], [23], [24], little is known about the molecular basis of Fe(II) oxidation: to date most genetic and biochemical studies have been conducted on the acidophilic bacterium *Acidithiobacillus ferrooxidans* [25], and the anoxygenic photosynthetic organisms *Rhodobacter sp.* strain SW2002 [26], and *Rhodopseudomonas palustris* [27]. These studies have led to the discovery of various proteins that are implicated in the enzymatic oxidation of Fe(II), however, proteins with an active role in microaerophilic Fe(II) oxidation by chemolithoautrophic bacteria at circumneutral pH have not been identified to date.

We have conducted a functional annotation of the genome of *Mariprofundus ferrooxydans* PV-1 with the aim of gaining insights into its phylogeny, physiology, and biochemistry. Comparative genomic analyses including genomes from other FeOB were used to define genomic commonalities between these phylogenetically and ecologically distinct neutrophilic Fe(II) oxidizing bacteria.

## Results and Discussion

### Phylogenetic context

**Figure 2:**
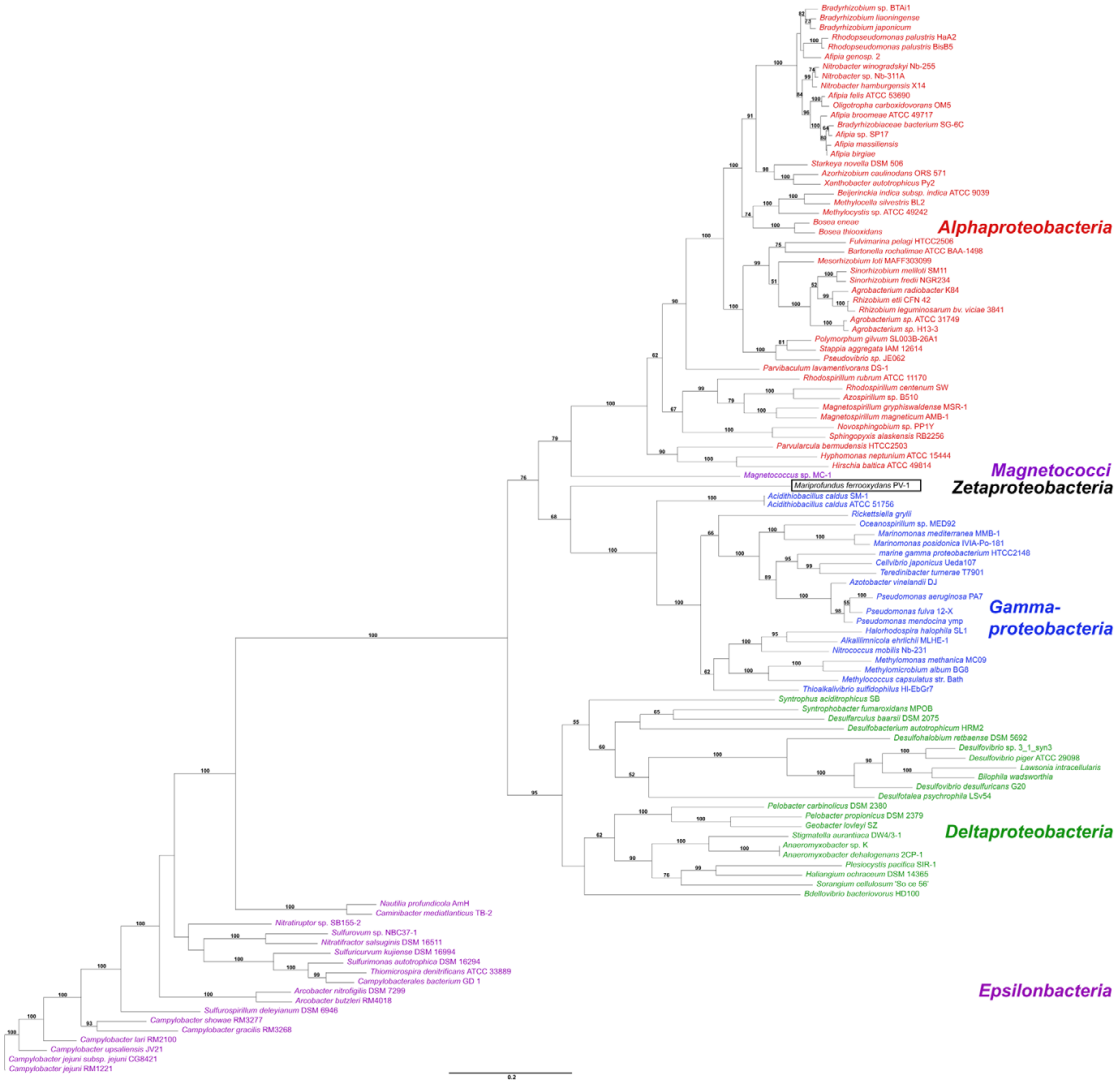
Phylogenetic placement of PV-1. Maximum-likelihood tree of ten proteins considered evolutionarily conserved: FusA, GyrB, IleS, LepA, LeuS, PyrG, RecA, RecG, RplB, RpoB [72]. *Mariprofundus ferrooxydans* PV-1 branches out as a distinct class within *the Proteobacteria* and appears most closely related to the *Magnetococci* subdivision, which only comprises one sequenced genome, *Magnetococcus* sp. MC-1, to date.

A previous phylogenetic analysis based on comparisons of the 16S rRNA gene, as well as GyrB and RecA proteins indicated that *M. ferrooxydans* did not belong to any of the recognized classes of *Proteobacteria* (Fig. S1) [1]. Analysis based on an amino acid sequence tree of ten concatenated conserved proteins (Fig. 2) supports these previous analyses and further demonstrates that PV-1 belongs to a new class within the *Proteobacteria*.

### General genome organization and content

The *Mariprofundus ferrooxydans* PV-1 draft genome sequence consists of 32 scaffolds. It comprises 2,867,087 bp with an average G+C content of 54% and has 2,866 protein coding sequences (CDSs). A mathematical model by [28] of the draft predicts the genome to include ∼98.5% of all CDSs, thus implying only ∼44 genes are missing. PV-1 carries 6 phage integrases and 21 transposases. The transposases are distributed relatively evenly across the genome scaffolds and are typically located next to genes with higher or lower G+C content compared to the genome average, required for signal transduction mechanisms, posttranslational modification, and cell motility, suggesting that some of the genes encoding these functions were obtained via lateral gene transfer (LGT).

One phage gene cluster consists of 32 CDSs and is flanked by a transposase (SPV1_02953) and three hypothetical proteins located upstream (Fig. S2). The G+C content varies between 48%–60% across the gene cluster, with 19 genes at 2–10 higher G+C% and 7 genes at 2–10 lower G+C%. The phage gene clusters with most significant nucleotide sequence alignment scores across the entire cluster of all 32 genes are found in *Pseudomonas* phage MP29 (30% NAID), Bacteriophage D3112 (29% NAID), and *Sideroxydans lithotrophicus* ES-1 (24% NAID) indicating potential LGT events between these organisms. Considering the similarity of prophage sequences between PV-1 and ES-1, this prophage region may have provided a selective advantage to neutrophilic FeOB.

### Metabolic processes

#### Carbon acquisition and storage

*M. ferrooxydans* is capable of growth in a mineral salts medium with Fe(II) as an energy source and CO_2_ as a carbon source [1]. The genome contains two sets of ribulose bisphosphate carboxylase (RuBisCo) genes, including the large and small subunit Form IAq RuBisCo (SPV1_12797, SPV1_12802) and a Form II RuBisCo (SPV1_04963). Both, Form I and Form II RuBisCo genes are located in typical gene clusters containing the two RuBisCo activation proteins CbbQ (SPV1_12807, SPV1_04958) and CbbO (SPV1_12812, SPV1_04953). Form IAq RuBisCo appears predominantly in obligate chemolithotrophs and functions best in niches with medium to low CO_2_ concentrations (0.1–1%) and O_2_ present [29]. Form IAq RuBisCo is not associated with carboxysomes and carbon concentrating mechanisms [29], however, it is not clear if that necessarily implies that organisms, which solely contain form IAq RuBisCo, are not capable of building carboxysomes. Form II RuBisCo proteins have a low discrimination threshold against O_2_ as an alternative substrate, poor affinity for CO_2_, and therefore potentially take over when the organism moves to a high-CO_2_ (>1.5%) and low-O_2_ environment [29]. It has been suggested that Form II RuBisCo may be a more ancient type of enzyme and Form I RuBisCo therefore an aerotolerant descendant [30]. *Proteobacteria* that encode both Form I and II RubBisCo proteins include purple non-sulfur bacteria and certain chemoautotrophic bacteria; most of these organisms appear to be predominantly facultative anaerobes that are metabolically versatile and globally distributed [31], [32], [33]. At the Loihi Seamount, temperature differences between bottom water (4°C) and hydrothermal efflux (55°C) may create turbulent eddies in the water column, which would expose cells to oscillating anaerobic and microaerobic conditions, where CO_2_ levels are variable (ranging from 2 µM to 20 µM) and dependent on positioning within the chemocline interface [29], [34]. Utilization of Form I and II RuBisCo proteins could thus enable PV-1 to optimize the acquisition of carbon under a wider range of CO_2_ and O_2_ concentrations in this dynamic system.

PV-1 also has three carbonic anhydrase-encoding genes (SPV1_01467, SPV1_09083, SPV1_07931) predicted to function in the rapid conversion of CO_2_ to bicarbonate (typically ∼10^6^ reactions per second) [35]. Two gene homologs to *cmpB* (SPV1_06134) and *cmp*C (SPV1_06129), which were shown to function in an operon (*cmpABCD*) encoding for bicarbonate uptake in *Synechococcus* sp. strain PCC 7942 [36], are located on a large gene cluster (20 genes), which includes a predicted urea carboxylase (SPV1_06124). Urea carboxylase is known to catalyze the conversion of ATP, urea, and bicarbonate to ADP, phosphate, and urea-1-carboxylate. CmpB, cmpC, and urea carboxylase could be part of a carbon-concentrating mechanism (CCM), although neither *ccmKLMNOP*, *chpXY* nor *cmpABCD* operons are observed and no carboxysomes have ever been observed by TEM (Chan, unpublished data). The range of inorganic and organic carbon substrates appears to be rather narrow for *M. ferrooxydans* [1], however PV-1 possesses a predicted operon (SPV1_t10271, SPV1_10194, SPV1_10199, SPV1_10204, SPV1_10209, SPV1_10214, SPV1_10219, SPV1_10224, SPV1_10229, SPV1_10234, SPV1_, SPV1_10239) encoding for a phosphoenolypyruvate-dependent sugar phosphotransferase system (PTS), which is the major carbohydrate transport system in bacteria [37]. The PTS enzyme II is a fructose/mannose-specific transporter in PV-1 (SPV1_10229). Fructose metabolism requires 1-P-phosphofructokinase [38], which appears to be missing in the PV-1 genome, however, imported mannose-6-phosphate could be converted by *manA* (SPV1_07961) to fructose-6-phosphate, which may then enter glycolysis I. This raises the possibility that carbon could be acquired in the form of carbohydrates from the environment, which would allow PV-1 to grow mixotrophically, although such behavior has not yet been observed in previous experiments, but was also not tested for fructose or mannose [1], [37]. Mannose may otherwise be used in glycoproteins and glycolipids, *e.g.* proteins that extend into the extracellular space, such as are required during stalk formation, and integral membrane proteins.

The genome of *M. ferrooxydans* shows the organism's potential ability to acquire and potentially store carbon from various sources as well as genomic evolution to the highly dynamic hydrothermal vent environment at Loihi. It remains to be experimentally tested if PV-1 solely utilizes imported carbohydrates for membrane and/or stalk synthesis or if this organism is in fact a mixotroph. The latter would imply that the organism could acquire carbon even when CO_2_ fixation is not possible in the niche it resides, and thereby enhance its chance of survival although carbon storage does not appear to be encoded in the genome.

### Energy acquisition: Aerobic Fe(II) oxidation at neutral pH

#### Microaerophily

The aerobic oxidation of Fe(II) requires *M. ferrooxydans* to live at the anoxic-oxic interface where it can outcompete the abiotic oxidation of Fe(II) [39], thus PV-1 should be adapted to capture oxygen at very low concentrations. Additionally, in oxic environments Fe(II) may react with hydrogen peroxide that is generated through oxidative processes, to form highly reactive oxygen species (ROS) via Fenton chemistry [40]. Since ROS have the potential to cause oxidative damage to DNA, RNA, and proteins, bacteria require defense mechanisms to convert these compounds into oxygen and water. The PV-1 genome contains a cytochrome *cbb_3_* oxidase regulon (*ccoNOP*) (SPV1_10291, SPV1_10301, SPV1_10306). CcoQ does not appear to be encoded, however, lack of this gene was shown to have no apparent effect upon the assembly or activity of cytochrome *cbb_3_* oxidase [41]. *Cbb_3_*-type cytochromes are members of the heme-copper oxidase superfamily that have the highest affinity for O_2_ among all cytochrome oxidases involved in microaerobic respiration [42], [43]. Substrate affinities have been measured in very few organisms so far, however, the high degree of sequence conservation of the catalytic subunit CcoN in *cbb_3_*-type cytochrome oxidases and the exclusive bacterial gene expression patterns under microaerophilic conditions suggests that cytochrome *cbb_3_*-type share oxygen affinities in *M. ferrooxydans* that are likely to be similar to such measured in other microaerophilic microorganisms [43].

In addition, there are two distinct cytochrome *bd* quinol oxidases (SPV1_03663, SPV1_03668) in the genome. These enzymes are distinct from heme-copper terminal oxidases and can function as oxidases and O_2_-scavengers [44] with K_m_ values for O_2_ in the range of 3–8 nM reported for *E. coli* [45]. Cytochrome *bd* may also help to mitigate oxidative stress by protecting cells from reactive oxygen species [44]. The expression of these genes could allow growth in oxygen limited habitats, such as is required in the reducing environment of the Loihi hydrothermal vents.

Protection against free oxygen radicals inside the cell is provided in part by a superoxide dismutase (SPV1_10466), several peroxidases (SPV1_03628, SPV1_11291, SPV1_13092), and alkyl hydroperoxide reductases (SPV1_06464, SPV1_08671), which also encode for predicted antioxidant response. Interestingly, genes encoding catalase and glutathione reductase that are present in nearly all organisms that are exposed to oxygen, including microaerophiles, such as *S. lithotrophicus*, were not found in the PV-1 genome [46]. While catalase produces H_2_O and O_2_ during the breakdown of H_2_O_2_, peroxidase requires NADH, but only produces H_2_O. The use of several peroxidases may therefore also be favored over that of catalase and superoxide dismutase because peroxidase reactions do not yield O_2_, which - when released to the environment - could affect the sensitive redox balance of iron and exacerbate microaerophilic Fe(II) oxidation. The suite of genes involved in respiration under microaerobic conditions as well as oxygen radical defense display how performance in a low oxygen environment is supported in the genome.

#### Fe(II) oxidation model

All current models for microbial Fe(II) oxidation and reduction involve the coupling of electron transfer to iron in the cytoplasmic membrane, so that insoluble minerals precipitate outside the cell. In the case of Fe(III) reduction, this concept is referred to as extracellular electron transfer [47] and several key genes have been identified in *Shewanella oneidensis* and *Geobacter sulfurreducens* [48], [49]. Similarly, there have been various key genes identified as relevant for Fe(II) oxidation. These include the *pio* and *fox* operon in the phototrophic organisms *Rhodobacter* sp. strain SW2 and *R. palustris*, respectively [27], and *iro*, *cyc1*, *cyc2*, *cox* genes and *rus* in the acidophilic *A. ferrooxidans* [25], [50]. The diversity of environmental conditions, under which microbial Fe(II) oxidation and ferric iron (Fe(III)) reduction may be performed, gives rise to diverse physiological mechanisms, biochemical pathways, and gene families involved in this process. Conservation of gene families between different microbial groups involved in Fe(II) oxidation and Fe(III) reduction is absent in most cases, however, few homologs with variable - generally low - sequence identities among key genes are observed [51]. The PV-1 genome harbors more than 70 genes required for electron transport (identified with Pfam domains). Most redox carriers belong to the cytochrome family, however, there are no gene homologs to the above mentioned iron redox genes in the PV-1 genome.

**Figure 3:**
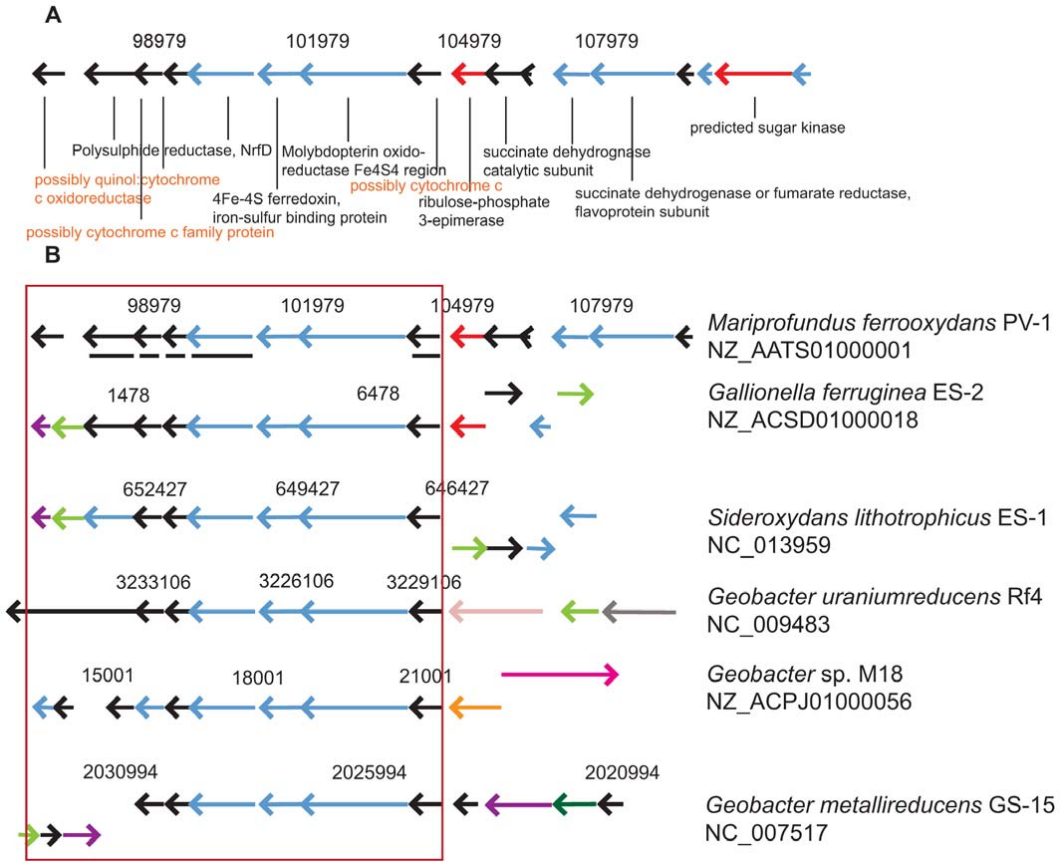
Iron oxidation candidate genes. A) Gene neighborhood of the extracted molybdopterin oxidoreductase protein (scaffold 1). Unlabeled genes are annotated as “hypothetical protein”. Putative functions of orange labeled genes were acquired via BLASTP search. B) Most similar orthologous neighborhoods were found in genomes from other metal oxidizing and reducing organisms in various *Proteobacteria* subdivisions. Most PV-1 genes within the red box (underlined) contain transmembrane helices indicating a location in either the inner or outer membrane and a potential role in electron transfer across membranes during Fe(II) oxidation. Coloring in A) and B) follows COG classification: blue = energy production and conversion; red: carbohydrate transport and metabolism; purple = general function prediction only; light green = posttranslational modification; rose = cell motility; grey = signal transduction mechanisms; orange = cell wall/membrane/envelope biogenesis; pink = inorganic ion transport and metabolism; dark green = coenzyme transport and metabolism. Source: IMG.

Heme-containing cytochromes with peroxidase activity were shown to be specifically expressed during Fe(II) oxidation in various organisms [26], [27], [50]. A protein significantly expressed in PV-1 cells oxidizing Fe(II) was extracted and identified as molybdopterin oxidoreductase Fe_4_S_4_ region (SPV1_03948). Protein topology prediction indicates a location of the encoded protein outside either membrane, possibly within the periplasm (Fig. S3). The gene neighborhood includes a cluster of 17 CDSs together with other cytochrome, succinate dehydrogenase, and ferredoxin encoding genes (Fig. 3A). Orthologous gene neighborhood comparison suggest most conserved gene content and synteny occurs with *G. capsiferriformans* and *S. lithotrophicus*, and to a lesser extent in *Geobacter uraniumreducens*, *Geobacter metallireducens*, and *Geobacter* sp. (Fig. 3B).

Molybdenum functions as a redox-active center, constituting a pterin cofactor in various enzymes involved in catalyzing oxygen atom transfer reactions to or from an electron donating/accepting substrate. Some of these enzymes facilitate the first step in redox reactions, (*e.g.* sulfite oxidase and assimilatory nitrate reductase), whereas other enzymes function as terminal respiratory oxidases, (*e.g.* DMSO reductase and biotin-*S*-oxide reductase) [52]. Electron transfer pathways proposed to specifically involve a molybdopterin oxidoreductase, include H_2_ oxidation during sulfate reduction [53] and the alternative complex III respiratory system [54]. Considering these examples and the protein isolation results (Barco *et al.*, in prep.), molybdopterin oxidoreductase and genes located in the same potential operon may play a significant role in the electron transport during Fe(II) oxidation.

**Figure 4:**
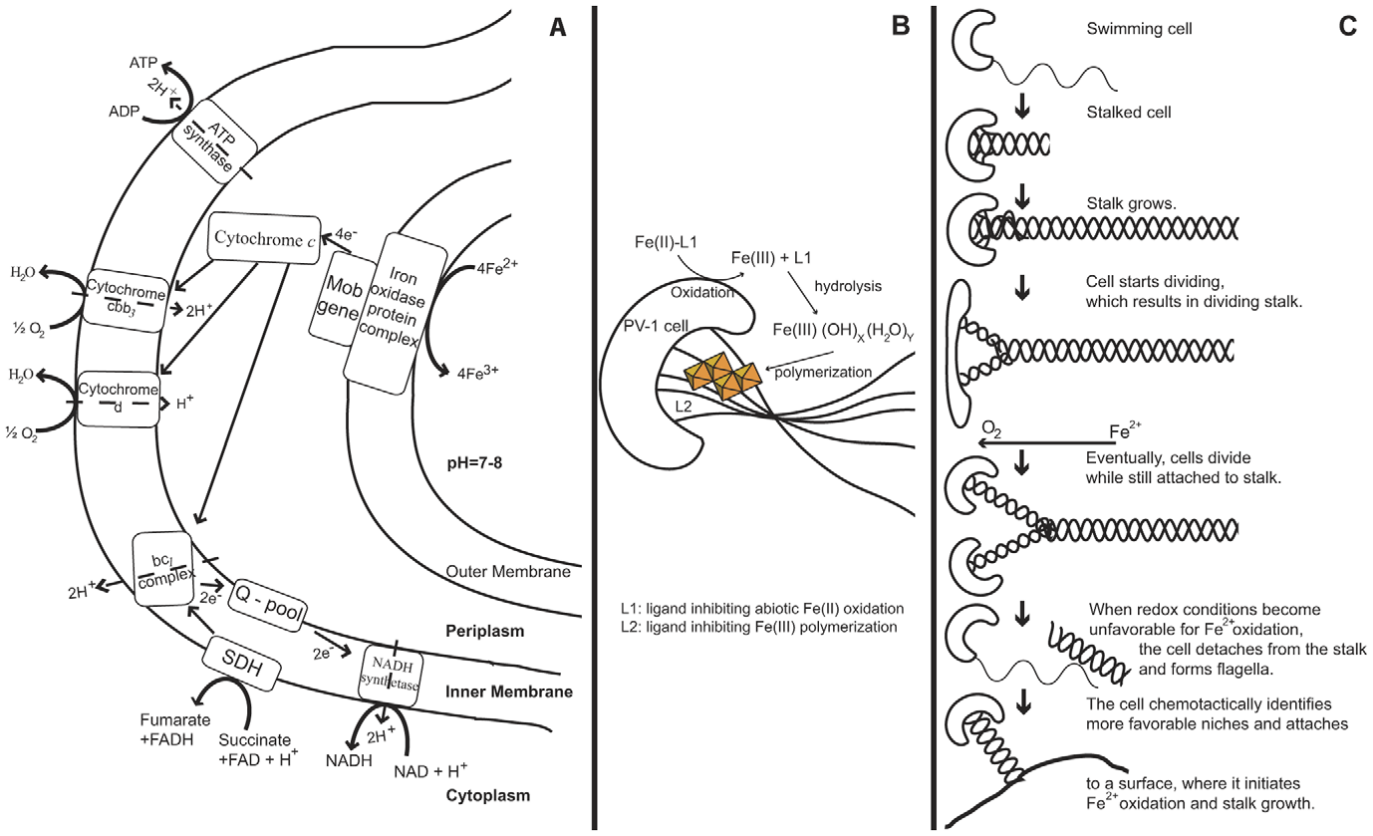
Conceptual iron oxidation model in relation to the life cycle in *M. ferrooxydans* PV-1. A: Proteins potentially involved in the energy acquisition via Fe(II) oxidation through the outer and inner membrane as predicted from genomic analysis. The “Mob gene”, possibly located in the periplasm, represents the experimentally identified molybdopterin oxidoreductase Fe_4_-S_4_ region (SPV1_03948), which was extracted under iron oxidizing conditions as mentioned earlier. Its function may include the shuttling of electrons between outer and inner membrane. B: Biologically formed iron oxides are stored in the stalk of PV-1 as edge-sharing Fe-O_6_ octahedral linkages as previously described in [76]. As the cell performs Fe(II) oxidation, it rotates, which results in a twisted, coiled stalk. C: Schematic of the life cycle in PV-1. The cell moves in the environment until it identifies conditions suitable for Fe(II) oxidation. The flagella are discarded and stalk growth initiated. As the cell divides, the stalk becomes bifurcated, and each cell continues to form a stalk that is initially half the width as observed by [12]. When O_2_ concentrations exceed the maximum tolerable by PV-1, the cell detaches from the stalk and forms flagella to move to a better-suited niche, where the life cycle starts over.

We propose an Fe(II) oxidation model as shown in Fig. 4A. The conversion of Fe(II) to Fe(III) may be catalyzed by an iron oxidase located in the outer membrane that is closely associated with a molybdopterin oxidoreductase Fe-S region located in the periplasm. The enzyme accepts electrons from ferrous iron and passes them on to an electron transport chain consisting of several oxygen sensitive cytochromes, which are predicted to be essential in the microaerobic environment PV-1 inhabits. Since the electrons obtained from the oxidation of Fe(II) with O_2_ are low potential electrons, reverse electron transport and the concurrent consumption of proton motive force are required for NADH synthesis.

#### Energy storage and life cycle

PV-1 exhibits a cell cycle from free-living motile cells to attached, stalk producing cells, which attach to substrates (glass surfaces, other Fe oxides, etc.), and produce Fe oxyhydroxides (Fig. 4C; also see [12]). Cells often undergo division and stalk bifurcation prior to detaching from attached substrate, when single cells enter a free living, motile stage. Motile cells are unattached to stalks and do not appear to oxidize Fe(II). During this motile phase cells are presumably using stored energy, like other obligate chemolithoautotrophic and photolithoautotrophic bacteria [55], [56], and may ferment stored organic compounds under anaerobic conditions to obtain ATP [56], [57].

Neither carboxysomes, nor poly-β-hydroxybutyric acid subcellular bodies have been identified in cells [58], and no genes (*ccmKLMNOP*, *chpXY*, *cmpABCD*) encoding carbon-concentrating mechanisms were identified in the genome. However, two genes encode glycogen/starch synthesis proteins (SPV1_03773, SPV1_01897) and glycogen and starch hydrolysis, *i.e.* usage of stored polysaccharides, are encoded by several amylases (SPV1_09118, SPV1_09123, SPV1_05592).

**Figure 5:**
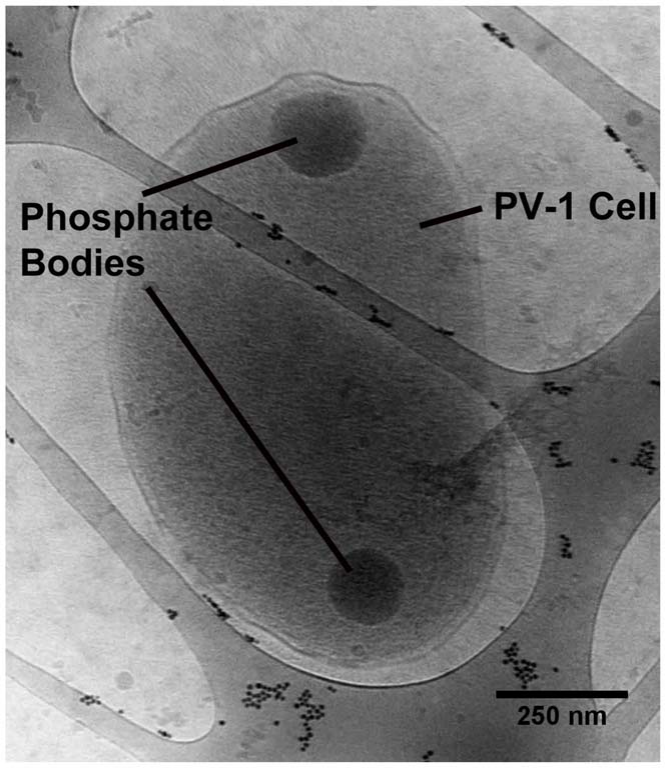
Cryo-TEM image of a *M. ferrooxydans* cell showing two polyphosphate bodies. Identification of polyphosphate bodies is based on electron density, electron dose tolerance, and shape as previously characterized by [58], who correlated these features to P electron spectroscopic imaging. Small dots on lacey carbon are 10 nm gold particles added to the sample.

Polyphosphate (poly P) has previously been proven to serve as energy and/or phosphate reservoir in *Thiobacillus* strain Q and *Accumulibacter phosphatis* [55], [59], [60]. Candidate genes involved in poly P synthesis were identified and poly P bodies were observed in PV-1 (Fig. 5). Several metabolic models for the use of poly P have been proposed, the consensus of which describes the uptake of inorganic phosphate (P_i_) via either low or high affinity P_i_ transporters (*e.g.* SPV1_07119, SPV1_07314, SPV1_07139) and conversion into poly P via ATP during conditions of carbon and energy excess in an aerobic environment [59]. Under anaerobic conditions, when the organism is in need of energy for the uptake of volatile fatty acids (VFAs), such as acetate and propionate that are stored as polyhydroxyalkanoates (PHAs), the phosphodiester bonds of the stored poly P are broken [59]. Enzymes shown to be involved in the degradation of poly P are polyphosphate:AMP phosphotransferase (SPV1_08276), which catalyzes the phosphorylation of AMP to ADP, and polyphosphate kinase (PPK) (SPV1_07169), which catalyzes ATP formation from ADP thereby enabling the use of poly P as energy source. Poly P may also be degraded into P_i_ for ATP production via V- and F-type ATPases (*e.g.* SPV1_13804, SPV1_13814, SPV1_13824) [59]. The source of the reducing power (NAD(P)H) required for PHA production may originate in the reverse electron transport chain through a *bc_1_* and NADH-Q oxidoreductase complex (*e.g.* SPV1_03858, SPV1_03863, SPV1_13739, SPV1_13744), as shown in *Thiobacillus ferrooxidans* [61]. Since the genome of PV-1 appears to encode for a complete set of genes required for the uptake and conversion of poly P to ATP, there is strong indication that the organism may use poly P as energy source as well as phosphate reserve during anaerobic conditions.

### Regulation and Signaling

*M. ferrooxydans* thrives best at low oxygen and high Fe(II) concentrations, however, the hydrothermal vent environment at Loihi is chemically heterogeneous and highly dynamic [34]. The organism therefore requires a chemotactic system that allows rapid sensation, signal transduction, and cell response in order to ensure flexibility and survival under suboptimal conditions. 9.35% of all CDSs in PV-1 are predicted to encode regulatory and signaling proteins, dominated by histidine kinases (43 CDSs) with various function domains, including PAS/PAC sensors, GGDEF/EAL, and multisensors. Other abundant functional genes include diguanylate cyclases (15), sensory box proteins (9), and (two-component) transcriptional regulators (19). In comparison, among other known neutrophilic Fe(II) oxidizers, regulatory and signaling genes comprise 10.61% (*G. capsiferriformans*) to 12.61% (*S. lithotrophicus*) of all CDSs, very similar to PV-1. In *Thiomicrospira crunogena* XCL-2, a sulfide oxidizer known to inhabit hydrothermal vents, 9.5% of all CDSs fulfill these functions, similar to strain PV-1. The primary role of PAS/PAC domains is the sensing of oxygen, redox, small ligand and overall cell energy level by binding redox or oxygen-sensitive ligands, such as heme and FAD in the cytosol [62]. PAS domains are understood to provide enhanced flexibility in adapting to complex redox environments [62]. EAL/GGDEF domain proteins catalyze the hydrolysis and the synthesis of cyclic diguanylate, an important intracellular signaling molecule, which in some species dictates the switch between attached and planktonic lifestyle via initiation of flagellar degradation and stalk formation [63]. All of these protein domains may provide an advantage when PV-1 detaches from its stalk and enters a stalk-free phase until it initiates Fe(II) oxidation and stalk formation in a better suited redox environment. Interestingly, the PV-1 genome draft harbors very few methyl-accepting chemotaxis protein-encoding (MCP) genes compared to other Fe(II) oxidizers as well as hydrothermal vent inhabiting organisms. There are only three CheY-like receiver proteins and one CheW protein. The family of MCP genes mediates chemotaxis to diverse signals, responding to changes in the concentration of attractants and repellents in the environment by altering swimming behavior. Each MCP is specific to a particular nutrient or toxin [64], therefore PV-1 may not require a large suite of MCPs if it follows a simple autotrophic lifestyle. There is also a full complement of flagellar genes (SPV1_01957-1967, SPV1_05769, SPV1_05579-05784, SPV1_07696-07701, SPV1_13924, SPV1_13954-13979) in the genome, which is consistent with the observation that PV-1 has a motile cell cycle stage (Fig. 4C). The mechanism by which it coordinates motility in response to chemical gradients remains to be biochemically established.

### Conclusions

Genome analysis of *M. ferrooxydans* PV-1 revealed first insights into the *Zetaproteobacteria* and disclosed candidate genes involved in inorganic and organic carbon acquisition, oxygen scavenging and defense, energy acquisition in the form of poly P, chemotaxis, and neutrophilic Fe(II) oxidation. The relative abundance of regulatory and signaling protein-encoding genes in PV-1 may be a reflection of the temporal and spatial heterogeneity of its hydrothermal vent habitat as previously described for the genome of *T. crunogena* XLC-2 [64]. The genomic potential predicting ability and tight regulation of mixotrophic growth, CO_2_ fixation under a variety of CO_2_/O_2_ concentration ratios and energy storage in phosphates as predicted from genomic potential show previously unknown degrees of flexibility that PV-1 may use to adapt to rapid redox chemistry changes at Loihi. Genes that have a potential role in Fe(II) oxidation show closest resemblance in gene content and synteny to organisms known to perform metal redox processes. *M. ferrooxydans* may be thus used as a model organism for future studies on neutrophilic, microaerophilic Fe(II) oxidation, which should address experimental verification of the suite of genes required for the enzymatically catalyzed conversion of Fe(II) to Fe(III).

Despite apparent genomic parallels to other FeOB from various *Proteobacteria* classes, relatively low amino acid sequence similarities between PV-1 and other *Proteobacteria* limit the ability to evaluate the evolutionary history of this organism's genome. The completion of this genome would allow more meaningful comparative genomics, verify or disprove speculations about missing functional genes, and provide insights into events in genome evolution, *e.g.* gene duplication and loss. Sequencing of additional *Zetaproteobacteria* strains will be useful to understand the metabolic and phylogenetic diversity within this recently discovered class and to examine the degree, to which the genomic potential is responsible for its dominance at the Loihi Seamount and possibly in other environments.

## Materials and Methods

### Organism and DNA preparation

*Mariprofundus ferrooxydans* PV-1 was isolated form an iron mat collected in 1996 associated with a cool (23°C), diffuse vent site at a depth of 1325 m at Loihi Seamounts described previously [65]. For DNA preparation, PV-1 was grown microaerobically on gradient plates. These petri plates contain 15 ml of artificial seawater medium overlaying an agarose/FeS layer that provides an iron source. Incubation was performed in a gas tight jar with a BBL Campypak™ (www.bd.com) that generates a microaerobic atmosphere [66]. Approximately 500 ml of late-log phase culture was concentrated by centrifugation and the pellet containing cells and Fe oxides was extracted for DNA using a MoBio PowerSoil DNA isolation kit (Mo Bio Laboratories, Carlsbad, CA), which yielded approximately 15 µg of good quality DNA.

### Genomic sequencing

Sequencing of the PV-1 genome was carried out at the J. Craig Venter Science Institute Joint Technology Center using conventional whole-genome shotgun sequencing. Two genomic libraries with insert sizes of 4 and 40 kb were made as described in [67], and resulted in 23,314 reads with an average read length of 951.13 bp at 7.61X coverage. Assembly of quality reads was done using the Celera Assembler [68]. The drafted genome sequence of *Mariprofundus ferrooxydans* PV-1 is available in a total of 32 gene scaffolds, which are available under GenBank accession numbers NZ_AATS01000001-AATS01000032.

### Sequence analysis and annotation

The DNA sequence was submitted to the JCVI Annotation Service and processed through JCVI's prokaryotic annotation pipeline. Included in the pipeline is gene finding with GLIMMER, Blast-extend-repraze (BER) searches, HMM searches, TMHMM searches, SignalP predictions, and automatic annotations from AutoAnnotate (www.jcvi.org/cms/research/projects/annotation-service/overview). Functional assignment, identification of membrane-spanning domains, determination of paralogous gene families, and identification of regions of unusual nucleotide composition were done as described [69]. Phylogenomic analysis was used to aid in functional predictions and alignments; phylogenetic trees were generated as described [69]. The annotated genome was submitted to the National Center for Biotechnology Information GenBank non-redundant database (NR) and the Integrated Microbial Genomes (IMG) database [70].

### Identification of genes

Genes involved in all addressed metabolic pathways were taken from the databases of Integrated Microbial Genomes (IMG, Version 3.3 February 2011; US Department of Energy Joint Genome Institute, supported by the DOE Office of Science). Manual annotation for final gene function assignments was performed using top gene homolog hits, which are based on pre-computed BLAST data from all IMG genomes and were identified on the basis of unidirectional and reciprocal hits with an e-value below 10^−2^. Functional gene groups were identified using cluster of orthologous group (COG) assignments and Pfam hidden Markov models provided by IMG. Alignments and phylogenetic determinations were performed using Geneious (Geneious Pro 4.8+; copyright © 2005–2010 Biomatters Ltd.) [71]. Universally conserved genes listed in [72] were concatenated in random order and aligned using the MAFFT Auto algorithm with a BLOSUM scoring matrix [73]. The phylogenetic tree was constructed with the PHYML algorithm using the JTT substitution model. Characterization of orthologous neighborhoods was conducted by searching for neighborhoods of roughly same sized orthologs (top COG hit) in all IMG genomes.

### Cryo-TEM sample preparation and analysis

*M. ferrooxydans* cells were cultured in petri plates for 1 day, mounted on a lacey carbon coated grid. The sample was blotted with filter paper, immediately plunge frozen in liquid ethane and stored in liquid nitrogen until analysis on a JEOL–3100 electron microscope equipped with a FEG electron source operating at 300 kV, an Omega energy filter, a Gatan 795 2Kx2K CCD camera, and cryo-transfer stage.

### Protein extraction

*M. ferrooxydans* cells were cultured microaerobically in liquid medium, which provided Fe(II) oxidizing conditions, as described in [66]. Proteins were extracted from an Fe(II) oxidizing PV-1 cell via a sodium dodecyl sulfate-polyacrylamide gel electrophoresis (SDS-PAGE) peptide analysis and heme staining as described in [74], [75]. The protein mentioned in this study, identified as molybdopterin oxidoreductase Fe_4_-S_4_ region, was found to be highly expressed under Fe(II) oxidizing conditions and yielded a strong band on the SDS-PAGE gel.

## Supporting Information

Figure S1 Maximum-likelihood phylogenetic tree showing the evolutionary placement of various strains of *Mariprofundus ferrooxydans* in the *Zetaproteobacteria* on the basis of 16S rDNA (reprinted from [1] with permission of the publisher).
(TIF)

Figure S2 Prophage gene cluster consisting of 32 CDSs on genome scaffold 21. Coloring is based on COG functionality: red = function unknown; purple = general function prediction only. Predicted functions of non-hypothetical genes are labeled respectively. BLASTP search revealed most significant alignment to gene clusters in *S. lithotrophicus* ES-1, *Pseudomonas* phage MP29, and Bacteriophage D3112.
(TIF)

Figure S3 Protein topology prediction of molydopterin oxidoreductase Fe_4_S_4_ region (SPV1_03948). Most of the amino acids are predicted to be hydrophilic and therefore located outside the membranes, possibly within the periplasm. The predicted signal peptide may help to transport this protein across membranes.
(TIF)
