# Supplementary figures and images for: Mariprofundus ferrooxydans PV-1 the First Genome of a Marine Fe(II) Oxidizing Zetaproteobacterium
Source: PLoS One. 2011 Sep 23;6(9):e25386. doi: 10.1371/journal.pone.0025386 (PMC3179512; doi:10.1371/journal.pone.0025386)

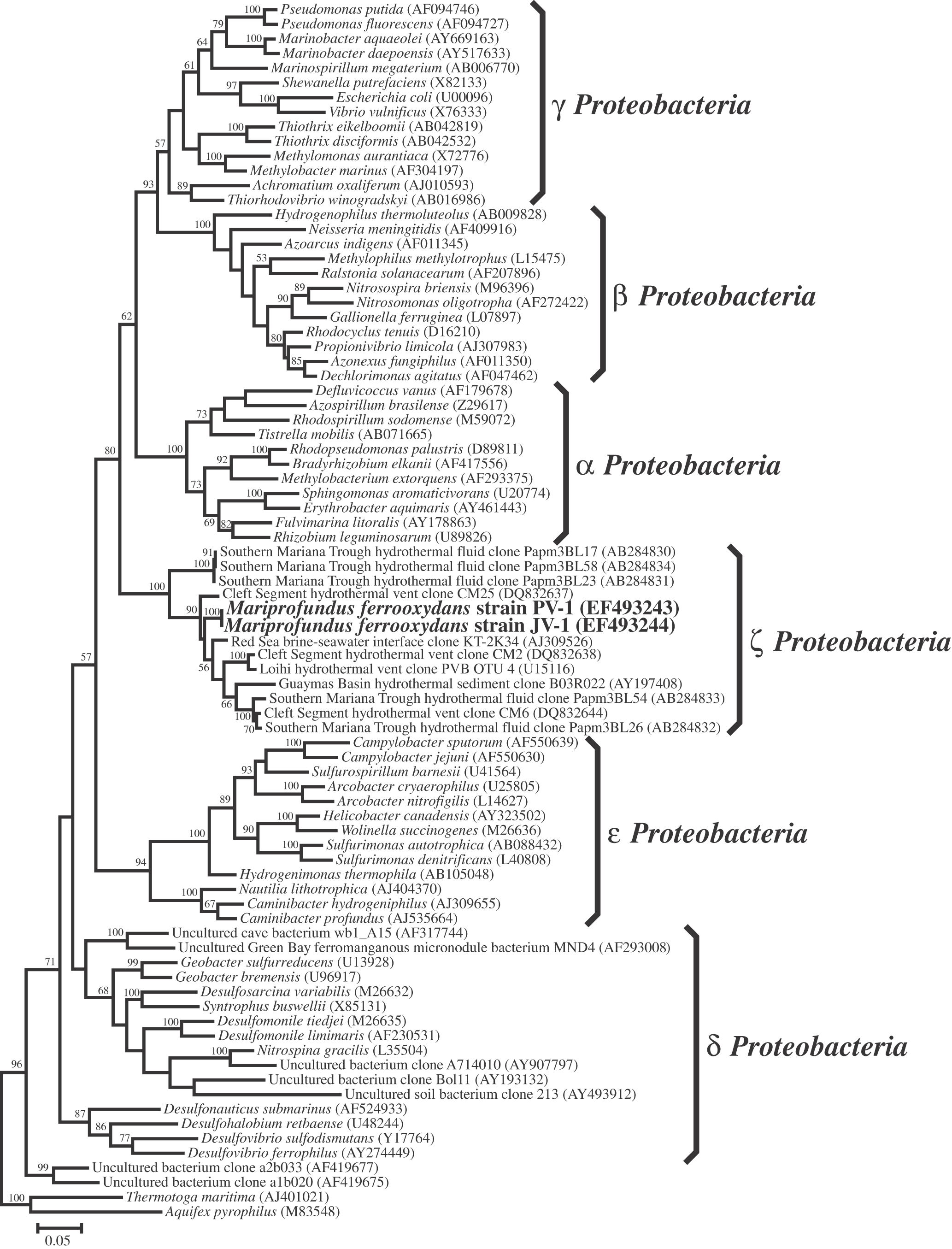

Supplement: Figure S1 — Maximum-likelihood phylogenetic tree showing the evolutionary placement of various strains of Mariprofundus ferrooxydans in the Zetaproteobacteria on the basis of 16S rDNA (reprinted from [1] with permission of the publisher). (TIF) [file pone.0025386.s001.tif]

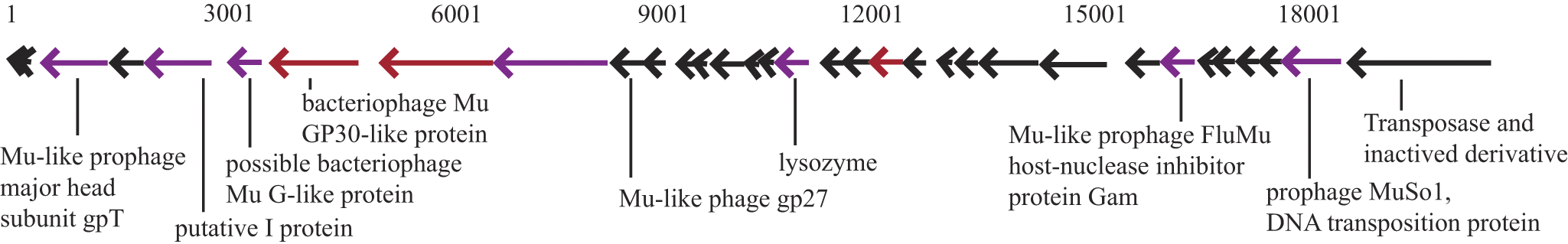

Supplement: Figure S2 — Prophage gene cluster consisting of 32 CDSs on genome scaffold 21. Coloring is based on COG functionality: red = function unknown; purple = general function prediction only. Predicted functions of non-hypothetical genes are labeled respectively. BLASTP search revealed most significant alignment to gene clusters in S. lithotrophicus ES-1, Pseudomonas phage MP29, and Bacteriophage D3112. (TIF) [file pone.0025386.s002.tif]

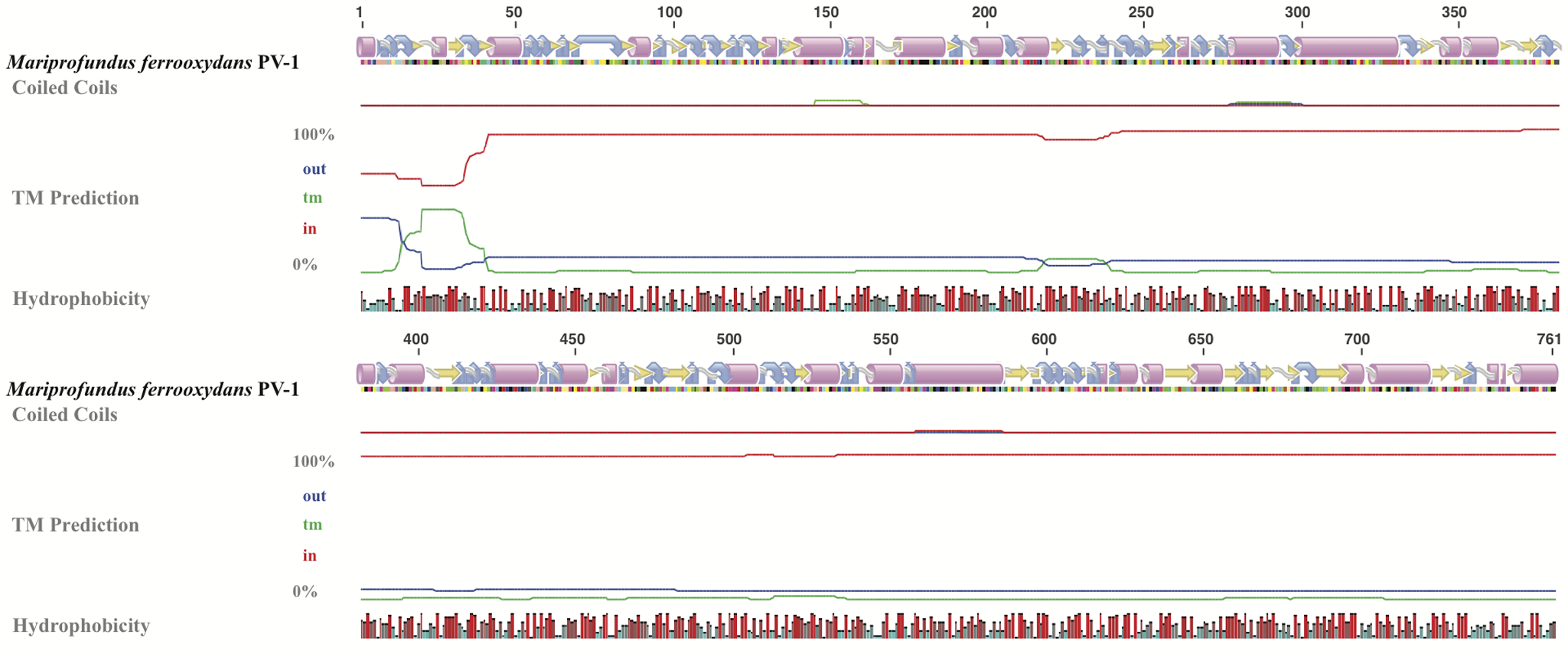

Supplement: Figure S3 — Protein topology prediction of molydopterin oxidoreductase Fe4S4 region (SPV1_03948). Most of the amino acids are predicted to be hydrophilic and therefore located outside the membranes, possibly within the periplasm. The predicted signal peptide may help to transport this protein across membranes. (TIF) [file pone.0025386.s003.tif]
